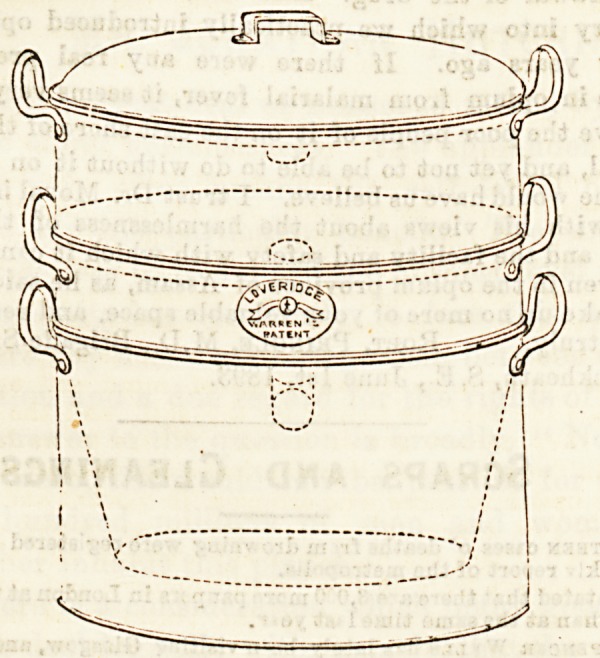# Warren's Cooking Pot

**Published:** 1893-06-17

**Authors:** 


### PRACTICAL DEPARTMENTS.

### WARREN'S COOKING POT.

Probably many of our readers will be familiar with this very useful cooking pot, which it is not too much to say is all but a necessity where sick-room cookery is concerned. Its advantages are very apparent, as will be seen by a glance at the accompanying diagrams, and the directions for its use are so simple that even the most inexperienced cook can find no difficulty in its management. The pot consists of three separate parts, "A is to contain the water, B the meat, game, curry, or whatever is to be cooked; C need be put on only when vegetables are to be cooked. The pipe on the side of B delivers the steam into the chamber C without having any access into the interior of B, the meat being cooked completely without any moisture but what it gives out as it becomes heated. The lid is made double to prevent the radiation of heat, and to condense the steam." The meat is placed in the pot B, without water, care being taken that the water in the saucepan A is high enough to touch the bottom of the pot. As a general rule an allowance as to time of a quarter of an hour for each pound of meat will be sufficient, of course reckoning the time from the moment when the water in the outer saucepan has began to boil. It is an undeniable fact that food prepared in this way is at once more nutritious and more digestible. The meat being kept entirely both from water and steam is cooked in its own vapour, and thus none of the nourishing juices are lost, as happens with the ordinary method. Soup can also be prepared in this pot, and will be found to be stronger and better, it being impossible on this plan for the nutritious ingredients to escape by evaporation. Fish kettles on the same principle can be had, but for general purposes the oval-shaped pot will probably be found the most convenient. The price varies according to size, from 10s. 6d. to 37s. 6d. A point we should not omit to mention is that the food can be kept hot in this fashion for a very long time, with no chance of its being spoilt. We have found the cooking pot simply invaluable in cases where feeble digestions have to be considered, and warmly recommend its use to all who have to cater for delicate appetites. We are indebted for the illustrations to the Wilson Engineering Company, 227, High Holborn, from whom these pots may be obtained.

## Figures and Tables

**Figure f1:**
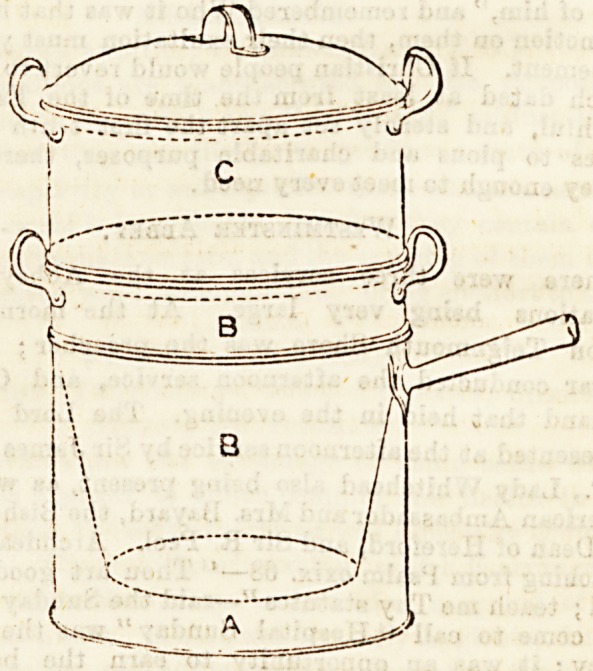


**Figure f2:**